# How did coronavirus disease 2019 affect autonomic balance in young individuals? Analysis by heart rate variability

**DOI:** 10.1590/1806-9282.20231789

**Published:** 2024-09-30

**Authors:** İmran Ceren, Fadime Bozduman Habip

**Affiliations:** 1 Ankara Abdurrahman Yurtaslan Oncology Education and Research Hospital, Department of Cardiology – Ankara, Turkey.

**Keywords:** COVID-19, Autonomic dysfunction, Heart rate determination

## Abstract

**OBJECTIVE:**

The aim of this study was to demonstrate the effect of coronavirus disease 2019 on the cardiovascular autonomic system using heart rate variability in young individuals.

**METHODS:**

The study was designed retrospectively by scanning the 24-h Holter electrocardiography records of patients who applied to the Ankara Abdurrahman Yurtaslan Oncology Education and Research Hospital Cardiology outpatient clinic. The study group consisted of 492 patients under the age of 40 years, who did not have additional comorbidities or medication use and had prolonged symptoms after coronavirus disease 2019 during the pandemic. The control group, including 401 patients, was determined during the pre-pandemic period (before December 2019). Heart rate variability parameters were evaluated by scanning the 24-h Holter electrocardiography records of the patients and compared with the non-coronavirus disease 2019 group.

**RESULTS:**

The median age of participants was 30 years. Standard deviation of normal RR intervals (SDNN) ≤100 ms was more prevalent in the study group (27 (6.7%) vs 73 (14.8%), p<0.001). In univariate logistic regression analysis, the presence of coronavirus disease 2019 [(OR 2.41, 95%CI 1.52–3.83), p<0.001] and age [(OR 1.04, 95%CI 1.01–1.07), p=0.016] had a significant effect on the probability of SDNN≤100. In multivariate logistic regression analysis, the presence of coronavirus disease 2019 [(OR 2.42, 95%CI 1.52–3.85), p<0.001] and age [(OR 2.42, 95%CI 1.52–3.85), p=0.016] had a significant effect on the probability of SDNN≤100. Frequency domain measures such as, high-frequency values were significantly higher in the study group (p=0.029). The study group’s low-frequency/high frequency ratio was significantly lower (p=0.019). The low-frequency/high-frequency ratio’s cut-off value was ≤2.77. for determining the differentiation between coronavirus disease 2019 positive and negative cases in the receiver operating characteristic analysis. The sensitivity rate was 80.7%. The area under the curve value is 0.546 (p=0.019).

**CONCLUSION:**

This study showed that coronavirus disease 2019 causes reduced heart rate variability and increased parasympathetic activity in young patients. This may explain the prolonged symptoms after coronavirus disease 2019 infection.

## INTRODUCTION

The coronavirus disease 2019 (COVID-19) caused a global pandemic following the initial epidemic in December 2019. The World Health Organization officially proclaimed the outbreak to be a pandemic on March 11, 2020^1^.

Heart rate variability (HRV) indices such as high-frequency (HF), the percentage of consecutive RR intervals (pNN50), and the root mean square difference of consecutive RR interval differences (RMSSD) indicate parasympathetic activity^2^. The autonomic nerve system’s general level of balance is indicated by the standard deviation of normal RR intervals (SDNN)^3^. SDNN values above 100 ms are deemed healthy. The Low Frequency/High Frequency (LF/HF) ratio is considered to stand for parasympathetic and sympathetic balance^2^. However, this issue is controversial in the literature. Billman argued that the LF/HF ratio cannot accurately measure sympathovagal balance^4^.

HRV has been extensively utilized to measure risk in many cardiac and noncardiac illnesses^5,6,7^.

Current research has shown a correlation between autonomic dysfunction and COVID-19. Reduced HRV is associated with a higher risk of cardiovascular mortality^8^. However, studies investigating autonomic function in young adults with COVID-19 by using HRV are limited. Soliński et al.^9^ compared young male subjects after COVID-19 with a control group without COVID-19 and found significant changes in HRV parameters associated with higher parasympathetic nervous system activity. On the contrary, Stute et al.^10^ showed that young adults had early autonomic changes that were characterized by higher HRV and resting muscle sympathetic nerve activity. Similarly, after COVID-19 in young adults, Freire et al.^11^ found decreased cardiac vagal control. In this regard, we aimed to evaluate the after-COVID-19 HRVs of individuals under the age of 40 years who had no additional risk factors and no history of chronic disease.

Our study differs from other studies in the literature investigating the effect of COVID-19 on HRV in that the participants were selected from individuals under the age of 40 and included a larger number of participants admitted throughout the entire pandemic. Moreover, the control group was selected from the pre-pandemic period in order not to randomly include asymptomatic COVID-19 patients.

So, we hypothesize that COVID-19 infection may cause impairment in the cardiovascular autonomic system in young individuals without causing major cardiovascular events. Deteriorations in HRV may lie at the root of increased orthostatic hypotension, palpitations, and similar complaints after the pandemic. By analyzing the 24-h rhythm Holter records of patients with COVID-19, it can be easily determined whether there is any deterioration in HRV data. The result of the study would highlight any possible derangements of HRV in young patients after COVID-19.

## METHODS

### Study design

The study was designed retrospectively and was confirmed by the Ankara Abdurrahman Yurtaslan Oncology Research and Education Hospital Clinical Research Ethics Committee (2023-11/112).

### Participants

Our study included patients who presented to the cardiology outpatient clinic with symptoms of palpitations that persisted for at least 4 weeks after being diagnosed with COVID-19 by polymerase chain reaction (PCR) between March 2020 and May 2022 during the pandemic period and had a 24-h Holter electrocardiography (ECG).

To avoid including individuals who may have had asymptomatic COVID-19 in the control group, the date before December 2019 was defined as the non-COVID-19 period.

Holter ECG records were detected in patients. Only patients under the age of 40 years were included in the study.

Patients with a history of additional comorbidities and chronic diseases such as heart failure, dysrhythmia, high blood pressure, coronary vascular disease, diabetes, heart valve failure, renal dysfunction, obesity, thyroid disease, malignancy, cerebrovascular disease, anemia, sleep apnea syndrome, and those who underwent ablation using antiarrhythmic and inhaler treatments that could affect HRV were excluded from the study by scanning through the electronic medical records.

### Data collection

Sociodemographic characteristics, comorbidities, laboratory results, smoking habits, 12 lead electrocardiograms, and transthorasic echocardiography findings of all patients were received through the electronic medical record, an online platform.

HRV was assessed through a 24-h ambulatory ECG monitoring system utilizing a multichannel electronic data recorder. This system enables the conversion and analysis of ECG information. The recorded ECG information from the Holter ECG recorder (BI9800TL, USA) was transferred to a computer equipped with dedicated software (BI, EcgLab, USA). Subsequently, the RR interval series from the recordings underwent frequency and time domain analysis throughout the 24-h duration. RR intervals were digitally filtered using the Biomedical Instruments EcgLab SW software (version 1.0.5.171016) and manually to eliminate ectopic beats and artifacts. For analysis, only series with more than 95% sinus beats were employed^12,13,14^. HRV was measured in the time domain as SDNN, RMSDD, and PNN50. HRV’s frequency domain indices, encompassing low frequency (LF, 0.04–0.15 Hz) and HF (0.15–0.40 Hz), were computed via spectral analysis over the entire 24-h period. The Fourier transform algorithm was utilized to calculate the spectrum analysis^12,14^.

### Statistical analysis

Descriptive statistics were used to summarize the acquired data. The normality of numerical variables was evaluated using Kolmogorov-Smirnov, Shapiro-Wilk, and Anderson-Darling tests. For comparing differences in categorical variables between groups, the Fisher’s exact test was employed for tables with expected observations less than 5 whereas the Pearson’s chi-square test was used for 2x2 tables with expected observations of 5 or more. The Fisher-Freeman-Halton test was utilized for RxC tables with expected observations of less than 5.

In comparisons between two independent groups, the independent samples t-test was used when numerical variables demonstrated a normal distribution, while the Mann-Whitney U test was applied in cases of a non-normal distribution.

Receiver operating characteristic (ROC) curve analysis was used. The area under the curve (AUC) value, accuracy, sensitivity, specificity, positive predictive value (PPV), and negative predictive value (NPV) of the LF/HF ratio were calculated. The optimal cut-off value, 95% confidence interval (CI) was determined. In this study, both univariate and multivariate logistic regression analyses were applied to determine the factors influencing SDNN≤100. Statistical analyses were conducted using Jamovi (Version 2.3.28) and JASP (Version 0.17.3) software, and the level of significance for the statistical analyses was set at 0.05 (p-value).

## RESULTS

For the study, the medical records of a total of 3,563 patients (1,894 patients from the pandemic period and 1,669 patients from the pre-pandemic period) were scanned. Among these patients, 2,367 patients over the age of 40 years and 303 patients with additional comorbidities that would affect HRV were excluded from the study. The final analyses included 492 patients with a history of COVID-19 and 401 age–gender-matched controls. The average age of the participants was 30 years. There were no statistical differences in general characteristics, laboratory parameters, or echocardiographic data between the study and control groups (Table 1).

**Table 1:** Comparison of general characteristics, laboratory and echocardiographic parameters between coronavirus disease 2019 patients and control group.

	Non-COVID-19 patients (control group) (n=401)	Post-COVID-19 patients (study group) (n=492)	p-value
Age (year)^§^	31.0 [17.0–40.0]	30.0 [18.0–40.0]	0.707*
Sex^‡^			
Female, n (%)	290 (72.3)	331 (67.3)	0.120**
Male, n (%)	111 (27.7)	161 (32.7)	
Smoking, n (%)	128 (31.9)	174 (35.4)	0.312**
SBP, mmHg^§^	120.0 [95.0–135.0]	120.0 [100.0–135.0]	0.262*
DBP, mmHg^§^	73.0 [60.0–85.0]	70.0 [60.0–85.0]	0.127*
Hemoglobin, (g/dL)^§^	13.4 [12.0–15.5]	13.2[12.1–15.3]	0.458*
White blood cells, (cells/μL)^†^	7.0 ± 1.4	6.8 ± 1.4	0.121***
Thyroid-stimulating hormone, (mIU/L)^§^	1.9 [0.3–4.4]	1.8 [0.4–4.8]	0.187*
Glucose, (mg/dL)^§^	85.0 [63.0–99.0]	85.0 [66.0–98.0]	0.797*
Creatine, (mg/dL)^§^	0.7 [0.5–1.1]	0.7 [0.4–1.0]	0.498*
Left ventricular ejection fraction, %^§^	64.0 [59.0–69.0]	64.0 [60.0–69.0]	0.256*
Left ventricular end-diastolic diameter, mm^§^	43.0 [39.0–44.0]	44.0 [39.0–45.0]	0.259*
Interventricular septum, mm^§^	10.0 [8.0–11.0]	10.0 [9.0–11.0]	0.670*
Posterior wall, mm^§^	9.0 [8.0–11.0]	9.0 [8.0–10.0]	0.522*
Left atrial diameter, mm^§^	33.0 [29.0–36.0]	32.0 [28.0–35.0]	0.658*

^‡^n (%);

^†^Mean ± standard deviation;

^§^median [min–max];

*Mann-Whitney U test;

**Pearson chi-square or Fisher’s exact test;

***Independent samples t-test.

SBP: systolic blood pressure; DBP: diastolic blood pressure.

Among time domain HRV parameters, SDNN, RMSDD, and PNN50 were similar between groups. The percentage of participants with SDNN ≤100 ms was evaluated. In the study group, the ratio of SDNN ≤100 ms was more common [27 (6.7%) vs 73 (14.8%), p<0.001]. Among frequency domain HRV parameters, LF values were similar between groups. However, in the study group, HF values were statistically higher (p=0.029), and the LF/HF ratio was statistically lower than in the control group (p=0.019) (Table 2).

**Table 2:** Comparison of heart rate variability parameters between coronavirus disease 2019 patients and control group.

	Non-COVID-19 patients (control group) (n=401)	Post-COVID-19 patients (study group) (n=492)	p-value
SDNN, ms^§^	145.0 [50.0–372.0]	146.5 [55.0–334.0]	0.960*
SDNN ≤100^‡^	27 (6.7)	73 (14.8)	<0.001**
RMSSD, ms^§^	38.0 [17.0–136.0]	39.0 [17.0–96.0]	0.087*
PNN50, %^§^	14.0 [1.0–44.0]	16.0 [1.0–53.0]	0.067*
HF, ms^2§^	337.0 [45.0–2137.0]	392.0 [24.0–2235.0]	0.029*
LF, ms^2§^	699.0 [116.0–2161.0]	733.5 [43.0–2307.0]	0.340*
LF/HF ratio^§^	2.0 [0.3–16.4]	1.8 [0.4–33.2]	0.019*

‡n (%),

§Median [min–max],

*Mann-Whitney U test,

**Pearson chi-square or Fisher’s exact test,

SDNN: standard deviation of NN intervals, RMSSD: root mean square of successive differences, pNN50: percentage of consecutive RR intervals, HF: high frequency, LF: low frequency. Statistically significant values are indicated in bold.

In univariate logistic regression analysis, the presence of COVID-19 (p<0.001) and age (p=0.016) had a significant effect on the probability of SDNN≤100. Accordingly, COVID-19 cases increased the odds of SDNN≤100 by a factor of 2.4 (OR 2.41, 95%CI 1.52–3.83), while a unit increase in age increased the odds of SDNN≤100 by 4% (OR 1.04, 95%CI 1.01–1.07). In multivariate analyses, COVID-19 status and age were included in the model together. In this model, the effect of both variables was significant (p<0.001 and p=0.016, respectively). Similarly, in multivariate analyses, the probability of SDNN≤100 increased 2.4-fold in COVID-19 cases (OR 2.42, 95%CI 1.52–3.85), whereas a unit increase in age increased the probability of SDNN≤100 by 4% (OR 1.04, 95%CI 1.01–1.07) (Table 3).

**Table 3:** Logistic regression analysis of factors influencing SDNN≤100 in participants.

Logistic regression predicting “SDNN ≤100”	Univariate logistic regression		Multivariate logistic regression	
	OR. [95%CI]	p-value	OR. [95%CI]	p-value
COVID: positive vs. negative	2.41 [1.52–3.83]	**<0.001**	2.42 [1.52–3.85]	**<0.001**
Age	1.04 [1.01–1.07]	**0.016**	1.04 [1.01–1.07]	**0.016**
Sex: male vs. female	0.88 [0.55–1.39]	0.571		
Smoking: yes vs. no	1.29 [0.84–1.98]	0.246		
TSH	1.12 [0.86–1.47]	0.390		
WBC	1.08 [0.93–1.25]	0.326		
Hemoglobin	0.99 [0.81–1.22]	0.946		

OR: odds ratio; CI: confidence interval; TSH: thyroid-stimulating hormone; WBC: white blood cells. Statistically significant values are indicated in bold.

**Figure 1:**
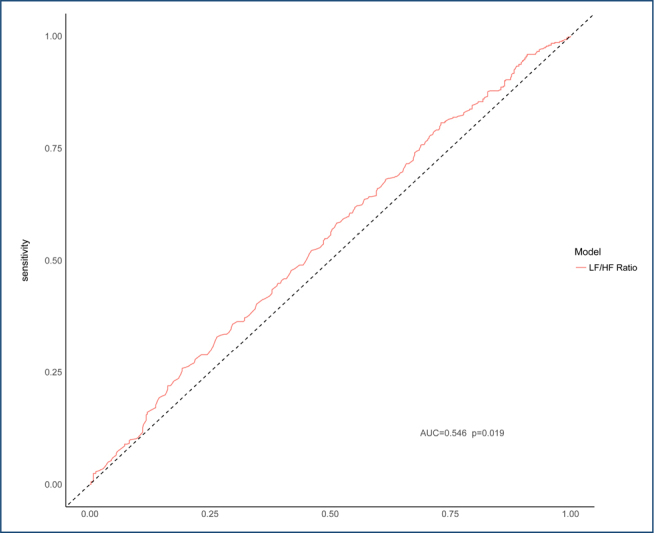
Receiver operating characteristic curve for low-frequency/high-frequency ratio in distinguishing between coronavirus disease 2019 positive and negative cases. AUC: area under the curve.

In the ROC analysis, the diagnostic characteristics of the LF/HF ratio were evaluated to determine the differentiation between groups. The cut-off value was ≤2.77 and the AUC value was 0.546 (p=0.019). The sensitivity rate was 80.7% and the specificity rate was 26.9%. The overall accuracy of the test was calculated to be 56.6%. The PPV was 57.5% and the NPV was 53.2% (Figure 1).

## DISCUSSION

The primary findings of our study include the following:

(i) compared to the control group, the COVID-19 group had a considerably increased percentage of patients with SDNN ≤100; and
(ii) a significant increase in parasympathetic activity, represented by elevated HF values and decreased LF/HF in the COVID group.

Nour et al. evaluated the relationship between HRV and Global Longitudinal Strain (GLS) in COVID-19, and the analysis showed that the decline in GLS and the decrease in SDNN and RMSDD showed a positive linear correlation^15^. This result may be associated with poor cardiovascular outcomes from COVID-19 when evaluated in parallel with the fact that there were significantly more people with SDNN≤100 in the COVID-19 group in our study and the statistical evidence that the decrease in SDNN with COVID-19 infection and age increases the risk.

Taş et al. conducted a detailed study on post-COVID-19 patients^16^. In this study, the initial HRV parameters of the patients were compared with the HRV parameters after 6 months, and the initial and 6th-month HRV parameters were compared with the control group, and unlike our study, no significant change was observed in any group comparison in the LF/HF ratio. Whereas, our investigation revealed a notable decline in the LF/HF ratio, and it was proven that being below the 2.7 cut-off value was significant in the diagnosis of COVID-19. This may be because our study included a larger number of participants and consisted only of symptomatic patients.

Unlike the findings in our study, Jesus et al.^17^ showed a significant increase in SDNN, pNN50, and RMSSD and a decrease in HF values in COVID-19 patients. The reason for these different results may be that the median age of individuals is 70 years. Age may significantly influence the relationship between HRV and inflammatory conditions. These findings, in light of our study, indicate that COVID-19 causes higher parasympathetic activity in young people compared to the elderly.

Another study compared young male subjects with mild symptoms after COVID-19 with a control group without COVID-19 and found similar results to our study by detecting significant differences in HRV parameters associated with higher parasympathetic nervous system activity^9^. HRV parameters were examined by Kaliyaperumal et al. in COVID-19^18^. They discovered an association between acute infection and parasympathetic dominance. These studies and ours are consistent in showing that post-COVID-19 patients have a persistent parasympathetic overtone, and our study supports the literature with its large number of participants.

Some studies have shown that hospital admissions decreased in specific patient groups during the pandemic^19^. Nevertheless, the number of patients in our study is large. This constitutes the strength of our study. However, our study has some limitations. It does not include specific patient groups that would restrict treatment^20^. Other limitations are that the admission times of the patients after COVID-19 infection are different, asymptomatic COVID-19 patients are not included in the study, its generalizability is limited since all comorbidities that may affect HRV are excluded, and patients’ long-term follow-up is not performed.

## CONCLUSION

This study showed that COVID-19 causes reduced HRV and increased parasympathetic activity in young patients. This may explain the prolonged symptoms after COVID-19.

## References

[1] Yin C, Li J, Wang Z, Zhi Y, Xu L (2023). Decreased heart rate variability in COVID-19. Intensive Care Res.

[2] Nunan D, Sandercock GR, Brodie DA (2010). A quantitative systematic review of normal values for short-term heart rate variability in healthy adults. Pacing Clin Electrophysiol.

[3] Evrengül H, Tanriverdi H, Dursunoglu D, Kaftan A, Kuru O, Unlu U (2005). Time and frequency domain analyses of heart rate variability in patients with epilepsy. Epilepsy Res.

[4] Billman GE (2013). The LF/HF ratio does not accurately measure cardiac sympatho-vagal balance. Front Physiol.

[5] Kurtoğlu E, Afsin A, Aktaş İ, Aktürk E, Kutlusoy E, Çağaşar Ö (2022). Altered cardiac autonomic function after recovery from COVID-19. Ann Noninvasive Electrocardiol.

[6] Carvalho TD, Norberto AR, Oliveira FR, Paiva LDS, Baracat EC, Soares JM (2022). Do heart rate variability indices present potential to predict late postmenopausal? A retrospective study. Rev Assoc Med Bras (1992).

[7] Almeida ÁD, Carvalho TD, Norberto AR, Figueiredo FWDS, Martinelli PM, Abreu LC (2021). Autonomic cardiac modulation in postmenopausal women with dry eye syndrome: a cross-sectional analytical study. Rev Assoc Med Bras (1992).

[8] Fang SC, Wu YL, Tsai PS (2020). Heart rate variability and risk of all-cause death and cardiovascular events in patients with cardiovascular disease: a meta-analysis of cohort studies. Biol Res Nurs. 2020;22(1):45-56.. Erratum in: Biol Res Nurs.

[9] Soliński M, Pawlak A, Petelczyc M, Buchner T, Aftyka J, Gil R (2022). Heart rate variability comparison between young males after 4-6 weeks from the end of SARS-CoV-2 infection and controls. Sci Rep.

[10] Stute NL, Stickford JL, Province VM, Augenreich MA, Ratchford SM, Stickford ASL (2021). COVID-19 is getting on our nerves: sympathetic neural activity and haemodynamics in young adults recovering from SARS-CoV-2. J Physiol.

[11] Freire APCF, Lira FS, Morano AEVA, Pereira T, Coelho-E-Silva MJ, Caseiro A (2022). Role of body mass and physical activity in autonomic function modulation on post-COVID-19 condition: an observational subanalysis of Fit-COVID study. Int J Environ Res Public Health.

[12] Catai AM, Pastre CM, Godoy MF, Silva ED, Takahashi ACM, Vanderlei LCM (2020). Heart rate variability: are you using it properly? Standardisation checklist of procedures. Braz J Phys Ther.

[13] Dias Carvalho T, Marcelo Pastre C, Claudino Rossi R, Abreu LC, Valenti VE, Marques Vanderlei LC (2011). Geometric index of heart rate variability in chronic obstructive pulmonary disease. Rev Port Pneumol.

[14] Carvalho TD, Pastre CM, Godoy MF, Fereira C, Pitta FO, Abreu LC (2011). Fractal correlation property of heart rate variability in chronic obstructive pulmonary disease. Int J Chron Obstruct Pulmon Dis.

[15] Nour A, Fouad M, Salam ZA (2023). Evaluation of cardiovascular autonomic dysfunction in symptomatic post-COVID-19 patients using the heart rate variability (HRV) and detection of subtle LV dysfunction using 2D global longitudinal strain (GLS). Int J Cardiovasc Imaging.

[16] Taş S, Taş Ü (2023). Effects of COVID-19 on the autonomic cardiovascular system: heart rate variability and turbulence in recovered patients. Tex Heart Inst J.

[17] Jesus P, Zangirolami-Raimundo J, Miranda JA, Sorpreso ICE, Raimundo RD (2023). Autonomic heart rate modulation in patients with coronavirus disease 2019 in mechanical ventilation. Rev Assoc Med Bras (1992).

[18] Kaliyaperumal D, Rk K, Alagesan M, Ramalingam S (2021). Characterization of cardiac autonomic function in COVID-19 using heart rate variability: a hospital based preliminary observational study. J Basic Clin Physiol Pharmacol.

[19] Parada LRC, Turri JAO, Helena Costa V, Vieira IB, Baracat EC, Soares JM (2023). Non-oncological gynecological diagnoses in a women’s health care service during the pandemic caused by the severe acute respiratory syndrome coronavirus 2. PLoS One.

[20] Soares JM, Sorpreso ICE, Motta EV, Utiyama EM, Baracat EC (2020). Gynecology and women’s health care during the COVID-19 pandemic: patient safety in surgery and prevention. Clinics (Sao Paulo).

